# Fish oil supplementation and risk of incident systemic lupus erythematosus: a large population-based prospective study

**DOI:** 10.1186/s12937-024-00965-x

**Published:** 2024-06-12

**Authors:** Yancong Chen, Zhilan Li, Yinyan Gao, Boya Xu, Weiru Zhang, Irene X.Y. Wu

**Affiliations:** 1 Changsha Center for Disease Control and Prevention, Changsha, 410004 Hunan China; 2 Department of Epidemiology and Health Statistics, Xiangya School of Public Health, Central South University, No. 172, Tongzipo Road, Yuelu District, Changsha, 410006 Hunan China; 3 Department of Rheumatology and Immunology, Xiangya Hospital, Central South University, Changsha, 410008 Hunan China; 4 Department of General Medicine, Xiangya Hospital, Central South University, No. 87 Xiangya Road, Kaifu District, Changsha, 410008 Hunan China; 5 Hunan Provincial Key Laboratory of Clinical Epidemiology, Central South University, Changsha, 410006 Hunan China

**Keywords:** Systemic lupus erythematosus, Fish oil, UK Biobank, Cohort study

## Abstract

**Background:**

Although fish oil has been considered to have an anti-inflammatory effect and has been proven to play a beneficial role in the incidence of numerous diseases, the association between fish oil supplementation and the risk of systemic lupus erythematosus (SLE) is still unknown. This study aimed at evaluating the correlation between fish oil use and incident SLE in a large population-based prospective cohort.

**Methods:**

390,277 participants without SLE at baseline from the UK Biobank were enrolled. Fish oil use was ascertained through a touchscreen questionnaire at baseline. The incidence of SLE was identified by the International Classification of Diseases version 10 code in medical records or self-report. Cox proportional hazard models were employed to estimate the association between fish oil use and SLE risk.

**Results:**

Fish oil users accounted for 31.47% of participants. During a median follow-up duration of 11.57 years, 141 participants without fish oil use (4.56/100 000 person-years) and 68 participants with fish oil use (4.78/100 000 person-years) developed SLE. In four models with adjustments for different amounts of confounders, there was no significant difference in the risk of SLE between fish oil users and fish oil non-users (all *p*-values > 0.05). In subgroup analyses, we found that fish oil supplementation was associated with a lower risk of SLE among females with ultraviolet radiation ≥ 3 h/day (hazard ratio: 0.63, 95% confidence interval: 0.40–0.98), which turned insignificant after further adjustment for female-related factors and sun protection measures.

**Conclusions:**

No significant association between fish oil use and overall incident SLE was observed, except in females exposed to prolonged ultraviolet radiation. Subgroup analysis suggested that females exposed to prolonged ultraviolet radiation might benefit from fish oil supplementation in terms of preventing SLE, but it needs to be confirmed in further studies.

**Supplementary Information:**

The online version contains supplementary material available at 10.1186/s12937-024-00965-x.

## Background

Systemic lupus erythematosus (SLE) is a classical autoimmune disease characterized by multiple positive autoantibodies and multi-organ dysfunction, with an increasing incidence and prevalence worldwide over the past decades [1]. It was reported that patients with SLE had a 2.6-fold higher all-cause standardized mortality ratio than the general population [2]. Moreover, affecting mainly women of reproductive age [1], SLE is a prominent cause of death among young women, ranking 10th in females aged 15–24 years in the United States and among the top 20 in females aged 10–54 years in Mexico [3, 4]. Although the pathogenesis of SLE is not fully understood, it has been recognized that excessive inflammatory responses and autoimmune activation, in the context of genetic factors and environmental triggers, are the central events in the pathophysiology of SLE [5]. Thus, understanding more adjustable risk factors for SLE is crucial for preventing the incidence of SLE.

Polyunsaturated fatty acids (PUFAs) can be classified into two primary categories, omega-3 (ω-3) PUFAs and omega-6 (ω-6) PUFAs, according to their chemical structure [6]. In general, ω-3 PUFAs have an anti-inflammatory property, thereby playing a protective role in a variety of diseases, including autoimmune diseases, while ω-6 PUFAs are considered to be pro-inflammatory factors and have harmful effects on body health [6, 7]. Fish oil is rich in ω-3 PUFAs, primarily docosahexaenoic acid (DHA) and eicosapentaenoic acid (EPA), and is thereby usually used as a dietary supplement for ω-3 PUFAs [8]. An increasing number of studies have suggested the inverse association of fish oil or ω-3 PUFAs supplementation with the risk of many diseases involving different systems, including dementia [9–11], liver disease [12], coronary heart disease in individuals with diabetes or prediabetes [13], chronic kidney disease [14], and type 2 diabetes [15] using a large population-based prospective cohort from the United Kingdom Biobank (UKB).

Also, fish oil or ω-3 PUFAs supplementation is shown to be associated with lower incidences of autoimmune diseases [16]. A Swedish prospective cohort study of 32,332 women showed that long-term intake of dietary ω-3 PUFAs was significantly associated with a lower risk of incident rheumatoid arthritis [17], although another American prospective cohort study of 80,551 women reported no significant association between intake of dietary ω-3 PUFAs and incident rheumatoid arthritis [18]. A case-control study suggested that regular fish oil use was inversely correlated with the risk of multiple sclerosis [19]. Similarly, a UKB study demonstrated the negative association of habitual fish oil supplementation with the incidence of inflammatory bowel diseases [20]. These diseases share the common pathophysiology of overactive inflammation, implying that fish oil supplementation may has a potential beneficial effect on the incidence of diseases involving inflammation.

Evidence from preclinical studies indicated a promising role of fish oil supplementation and ω-3 PUFAs in delaying the onset and progression of SLE in mouse models [21, 22]. Furthermore, evidence from a systematic review and clinical trials also suggested that ω-3 PUFAs and fish oil supplementation have a promising therapeutic effect in improving specific clinical symptoms, reducing disease activity, and ameliorating organ involvement among patients with SLE [8, 23, 24]. However, we did not identify any human-level evidence regarding the relationship between fish oil supplementation and incident SLE.

Hence, this present study aimed to examine the association between fish oil use and the incidence of SLE using a prospective cohort with a large sample size and long-term follow-up from the UKB and provide further evidence for the preventives of SLE.

## Methods

### Study design and population

The UKB is a large population-based prospective cohort study conducted in 22 assessment centers throughout the UK. Between 2006 and 2010, the UKB recruited more than 500,000 participants aged 40–69 years from the general population. Baseline demographic and lifestyle information was collected via a self-completed touchscreen questionnaire, and anthropometric measurements were taken by trained staff. A detailed description of the UKB project is reported elsewhere [25]. The UKB research was approved by the Northwest Multicenter Research Ethical Committee (reference number: 11/NW/0382). All participants provided written informed consent for the study.

**Fig. 1:**
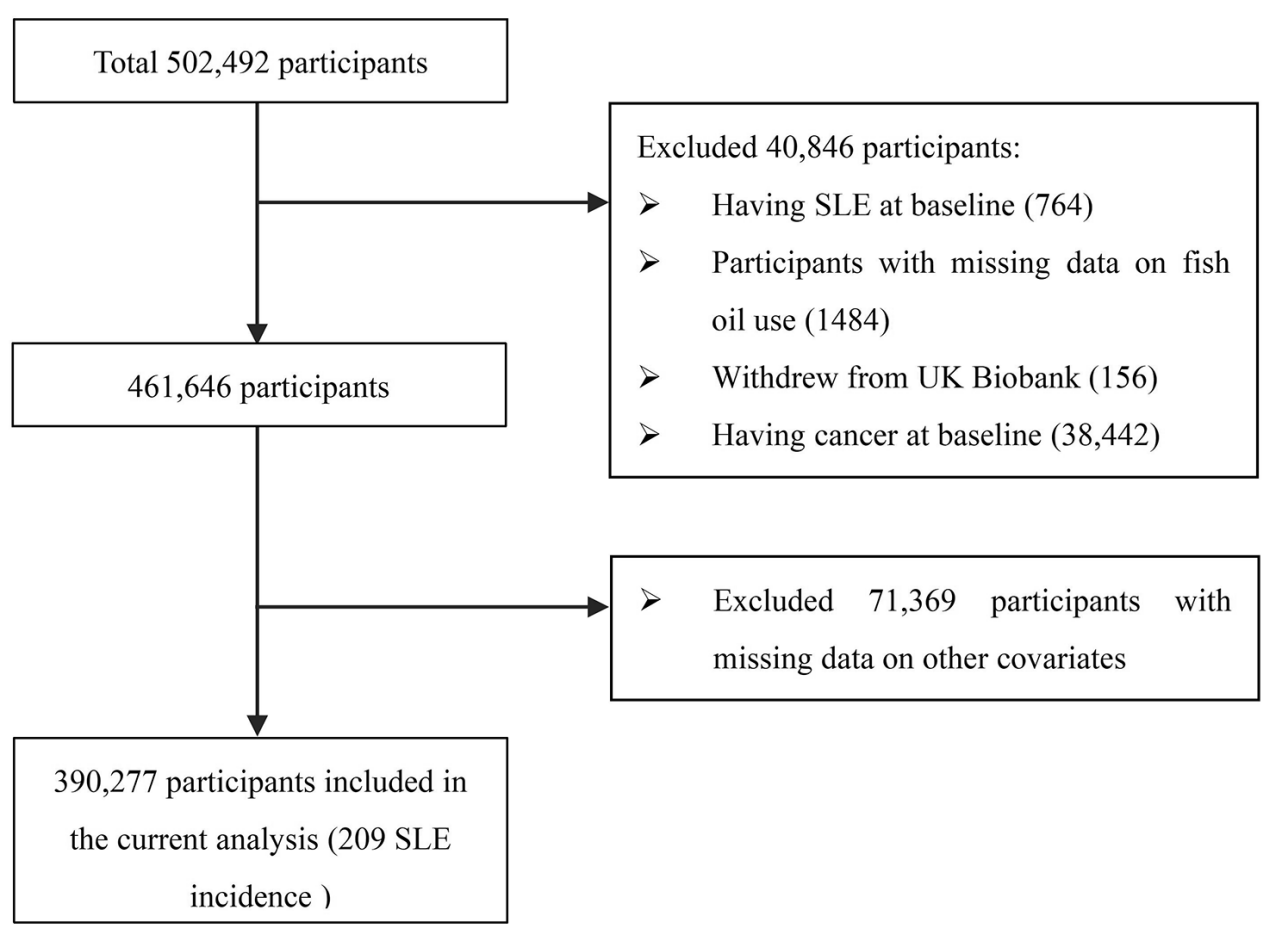
Flowchart for the selection of the analyzed study sample from the UK Biobank

Data from 502,492 participants were available for our study. Participants with SLE at baseline were excluded (*n* = 764). We also excluded participants with cancer at baseline (*n* = 38,442), those who withdrew from UKB during the follow-up (participants have withdrawn consent for future data linkage, *n* = 156), and those with missing data on fish oil use (*n* = 1,484). In this process, 461,646 participants were included. After excluding participants with missing data on other covariates (e.g. smoking status, drinking status, physical activity, history of diseases, *n* = 71,369), we included 390,277 participants in our analysis in the end. Details of the study sample selection are shown in Fig. 1. Detailed information on the number of missing covariates is shown in Supplementary Table S1.

### Ascertainment of fish oil use and covariates

In the baseline assessment, participants were asked, “Do you regularly take any of the following supplements?” through a touchscreen questionnaire. Multiple dietary items were incorporated, including fish oil use. We classified participants into fish oil user and fish oil non-user groups according to whether they selected the “fish oil (including cod liver oil)” item.

Information about other covariates was also obtained in the baseline assessment from touchscreen questionnaires or by linking to hospital inpatient records. Age, sex, race, location of assessment centers, body mass index (BMI), education, Townsend deprivation index (TDI), smoking status, alcohol drinking status, physical activity, dietary intake, vitamin supplementation use, mineral supplementation use, the use of nonsteroidal anti-inflammatory drugs (NSAIDs), history of hypertension, history of diabetes, history of hyperlipemia and ultraviolet (UV) radiation were included.

Education level was categorized as higher (College/university degree or other professional qualification), upper secondary (A levels/AS levels or equivalent), lower secondary (O-levels or Certificate of Secondary Education or equivalent), or other. BMI was calculated as the weight (kg) divided by height squared (m^2^), and classified into four groups based on the World Health Organization standards [26]. Socioeconomic status was reflected by using the TDI, which was calculated based on postcode-specific information on the percentages of unemployment, overcrowded households, people with no car ownership, and non-homeowners [27]. The higher the score, the higher the level of deprivation. According to the healthy physical activity recommendations from the World Health Organization [28], we classified participants into two groups based on the total time spent in moderate physical activity or vigorous physical activity in minutes each week (dichotomous variable): less than 150 min, 150 min or more per week. NSAIDs use included the use of aspirin, ibuprofen, or paracetamol. The histories of hypertension, diabetes, and hyperlipemia were defined according to self-reported information or hospital in-patient records, and detailed definitions of those diseases are shown in Supplementary Table S2. UV radiation was measured by asking, “In a typical day in summer, how many hours do you spend outdoors?” According to the definition of previous study [29], we divided participants into two groups (“<3 hours/day”, “≥3 hours/day”).

Dietary intake included 10 types of food (fruit, vegetables, whole grains, refined grains, fish, dairy, vegetable oils, processed meat, unprocessed meat, and sugar-sweetened beverages). We also constructed a healthy diet score based on the definition of ideal dietary component intake for cardiometabolic health [30]. One point was given if the intake goal was met. The detailed definitions of each type of food intake and intake goal were presented in Supplementary Table S3. The healthy diet score ranged from 0 to 10, with a higher diet score representing a healthier diet. The healthy diet score ≥ 5 was considered as an ideal diet, < 5 was considered a poor diet [30]. In analyses, we included healthy diet score as dichotomous variable (< 5, ≥ 5).

### Outcome ascertainment

The outcome of the study was the incidence of SLE. We used the data fields 131,894 and 131,895 in the UKB. These two data fields recorded all sources (primary care, hospital admission, death register, and self-report) of SLE and the first SLE occurrence date. The International Classification of Diseases version 10 (ICD-10) code M32 was used for the identification.

### Statistical analysis

The baseline characteristics of participants were described as means ± standard deviation (SD) for continuous variables or numbers (percentages) for categorical variables. The follow-up time was calculated from the baseline date to the date of the first SLE occurrence, death, lost to follow-up, or last update date of the linkages (30 November 2020), whichever occurred first. Death data were obtained by linking death registry records. Lost to follow-up was defined as the inability to follow an outcome because of departure from the UK or because a relative had reported death or national datasets indicated lost to follow-up.

The association between fish oil use and SLE incidence was explored by using Cox proportional hazard models, and the hazard ratios (HR) and 95% confidence intervals (CI) were calculated. The Schoenfeld residual method was used to test the proportional hazards assumptions for the Cox model. In our analyses, no violation of this assumption was observed. Four Cox models were built. Model 1 adjusted for age and sex. Model 2 adjusted for age, sex, race, location of assessment centers, BMI, education, TDI, smoking status, alcohol drinking status, physical activity, vitamin supplementation use, mineral supplementation use, NSAIDs use, history of hypertension, history of diabetes, history of hyperlipemia, and UV radiation. Model 3 further adjusted for fruit, vegetables, whole grains, refined grains, oily fish, non-oily fish, dairy, vegetable oils, processed meat, unprocessed meat, and sugar-sweetened beverages based on Model 2. Considering the complex interactions between dietary components, we further adjusted for the healthy diet score representing the overall diet quality in model 4 based on model 2.

We performed subgroup analyses to estimate the potential modification effect according to age (< 60, ≥ 60 years), sex (male, female), race (White, non-White), BMI (< 30, ≥ 30 kg/m^2^), current smoking status (yes, no), current drinking status (yes, no), physical activity (< 150, ≥ 150 min/week), vitamin supplementation use (yes, no), mineral supplementation use (yes, no), NSAIDs use (yes, no), history of hypertension (yes, no), history of diabetes (yes, no), history of hyperlipemia (yes, no), oily fish (< 1, ≥ 1 times/week), non-oily fish (< 1, ≥ 1 times/week), UV radiation (< 3, ≥ 3 h/day), and healthy diet score (< 5, ≥ 5). Potential modifying effects were assessed by modeling the cross-product term of the stratifying variable with fish oil use. As females are a high-risk group for SLE [31], to explore whether these stratifying variables have modifying effects in female group, we also conducted subgroup analysis and calculated *p* for interaction in the female group.

We performed a sensitivity analysis by excluding participants who developed SLE during the first two years of follow-up to test the robustness of our study and minimize the influence of reverse causation. Based on the previous evidence that showed some female factors related to the risk of SLE [32], we further adjusted for age at menarche (≤ 10 years, > 10 years), oral contraceptive use (yes, no), menopause status (yes, no, not sure), and hormone-replacement therapy used (yes, no) in the female group for sensitivity analysis. In addition, we further tested the robustness of the results by performing sensitivity analyses adjusting for the use of sun protection measures (never/rarely, sometimes, most of the time, always, do not go out in sunshine). The measuring methods of these five potential covariates (age at menarche, oral contraceptive use, menopause status, hormone-replacement therapy used, sun protection measures) are shown in Supplementary Table S4.

All analyses were performed using Stata (version 16) and R (version 4.1.1). Statistical significance was defined as a two-sided *p*-value < 0.05.

## Results

### Baseline characteristics

Our study included a total of 390,277 participants, of whom 122,829 (31.47%) reported habitually using fish oil at baseline. The baseline characteristics of the included participants categorized by fish oil use are shown in Table 1. Compared to fish oil non-users, fish oil users were older. Moreover, fish oil users had higher proportions of female, White, current drinkers, more physically active, and having a lower TDI, a normal BMI, as well as prolonged exposure to UV radiation. Fish oil users had lower proportions of being highly educated and current smokers. A greater proportion of participants took vitamin and mineral supplements and NSAIDs (including aspirin) among fish oil users. They also had a lower prevalence of diabetes but higher prevalences of hypertension and hyperlipidemia than fish oil non-users. Importantly, fish oil users had a higher healthy diet score as they consumed fruits, vegetables, oily fish, non-oily fish, whole grain, and vegetable oils more frequently, whereas they consumed refined grain, processed meat, and sugar-sweetened beverages less frequently.

**Table 1:** Baseline characteristics of participants in main analysis

Characteristics	Overall (*n* = 390,277)	Fish oil non-users (*n* = 267,448)	Fish oil users (*n* = 122,829)	*p*-value
Age (years)	56.14 ± 8.10	55.13 ± 8.18	58.34 ± 7.48	< 0.001
Sex				< 0.001
Male	182,804 (46.84)	127,891 (47.82)	54,913 (44.71)	
Female	207,473 (53.16)	139,557 (52.18)	67,916 (55.29)	
Race				< 0.001
White	372,437 (95.43)	254,713 (95.24)	117,724 (95.84)	
Non-White	17,840 (4.57)	12,735 (4.76)	5105 (4.16)	
Education				< 0.001
Higher	244,644 (62.68)	170,226 (63.65)	74,418 (60.59)	
Upper secondary	22,477 (5.76)	15,456 (5.78)	7021 (5.72)	
Lower secondary	65,764 (16.85)	44,650 (16.69)	21,114 (17.19)	
Others	57,392 (14.71)	37,116 (13.88)	20,276 (16.51)	
Townsend deprivation index	-1.45 ± 3.00	-1.37 ± 3.04	-1.63 ± 2.90	< 0.001
BMI (kg/m^2^)				< 0.001
<18.5	1936 (0.50)	1399 (0.52)	537 (0.44)	
18.5–24.9	129,605 (33.21)	87,402 (32.68)	42,203 (34.36)	
25-29.9	166,931 (42.77)	113,173 (42.32)	53,758 (43.77)	
≥ 30	91,805 (23.52)	65,474 (24.48)	26,331 (21.44)	
Smoking status				< 0.001
Current	39,517 (10.13)	29,743 (11.12)	9774 (7.96)	
Previous	135,152 (34.63)	88,523 (33.10)	46,629 (37.96)	
Never	215,608 (55.24)	149,182 (55.78)	66,426 (54.08)	
Alcohol drinking status				< 0.001
Current	362,739 (92.94)	247,907 (92.69)	114,832 (93.49)	
Previous	12,860 (3.30)	9042 (3.38)	3818 (3.11)	
Never	14,678 (3.76)	10,499 (3.93)	4179 (3.40)	
Physical activity (min/week)				< 0.001
< 150	161,429 (41.36)	116,203 (44.45)	45,226 (36.82)	
≥ 150	228,848 (58.64)	151,245 (56.55)	77,603 (63.18)	
Vitamin supplementation	124,255 (31.84)	54,396 (20.34)	69,859 (56.88)	< 0.001
Mineral supplementation	47,522 (12.18)	21,586 (8.07)	25,936 (21.12)	< 0.001
NSAIDs use	153,658 (39.37)	101,725 (38.04)	51,933 (42.28)	< 0.001
History of diabetes	19,386 (4.97)	13,638 (5.10)	5748 (4.68)	< 0.001
History of hypertension	110,060 (28.20)	73,238 (27.38)	36,822 (29.98)	< 0.001
History of hyperlipidemia	66,756 (17.10)	43,243 (16.17)	23,513 (19.14)	< 0.001
UV radiation (hours/day)				< 0.001
< 3	141,297 (36.20)	101,193 (37.84)	40,104 (32.65)	
≥ 3	248,980 (63.80)	166,255 (62.16)	82,725 (67.35)	
Fruit (servings/day)				< 0.001
< 1.0	34,613 (8.87)	27,704 (10.36)	6909 (5.62)	
1.0-2.9	210,775 (54.01)	148,743 (55.62)	62,032 (50.50)	
≥ 3.0	144,889 (37.12)	91,001 (34.03)	53,888 (43.87)	
Vegetable (servings/day)				< 0.001
<1.0	67,675 (17.34)	50,470 (18.87)	17,205 (14.01)	
1.0-2.9	288,753 (73.99)	194,808 (72.84)	93,945 (76.48)	
≥ 3.0	33,849 (8.67)	22,170 (8.29)	11,679 (9.51)	
Oily fish (times/week)				< 0.001
< 1	171,440 (43.93)	127,440 (47.65)	44,000 (35.82)	
1	148,760 (38.12)	97,682 (36.52)	51,078 (41.58)	
≥ 2	70,077 (17.96)	42,326 (15.83)	27,751 (22.59)	
Non-oily fish (times/week)				< 0.001
< 1	130,766 (33.51)	94,814 (35.45)	35,952 (29.27)	
1	195,620 (50.12)	130,920 (48.95)	64,700 (52.67)	
≥ 2	63,891 (16.37)	41,714 (15.60)	22,177 (18.06)	
Dairy (servings/day)				0.101
< 2	390,088 (99.95)	267,308 (99.95)	122,780 (99.96)	
≥ 2	189 (0.05)	140 (0.05)	49 (0.04)	
Whole grain (servings/day)				< 0.001
< 1.0	160,861 (41.22)	118,267 (44.22)	42,594 (34.68)	
1.0-2.9	174,848 (44.80)	113,634 (42.49)	61,214 (49.84)	
≥ 3.0	54,568 (13.98)	35,547 (13.29)	19,021 (15.49)	
Refined grain (servings/day)				< 0.001
≤ 2	321,689 (82.43)	216,219 (80.85)	105,470 (85.87)	
> 2	68,588 (17.57)	51,229 (19.15)	17,359 (14.13)	
Vegetable oils (servings/day)				0.006
< 2	300,342 (76.96)	206,151 (77.08)	94,191 (76.68)	
≥ 2	89,935 (23.04)	61,297 (22.92)	28,638 (23.32)	
Processed meat (servings/week)				< 0.001
≤ 1	268,174 (68.71)	180,437 (67.47)	87,737 (71.43)	
> 1	122,103 (31.29)	87,011 (32.53)	35,092 (28.57)	
Unprocessed red meat (servings/week)				< 0.001
≤ 2	62,585 (16.04)	43,932 (16.43)	18,653 (15.19)	
> 2	327,692 (83.96)	223,932 (83.57)	104,176 (84.81)	
Sugar-sweetened beverages consumer	319,562 (81.88)	222,504 (83.20)	97,058 (79.02)	< 0.001
Healthy diet score				< 0.001
<5	319,738 (81.93)	224,489 (83.94)	95,249 (77.55)	
≥5	70,539 (18.07)	42,959 (16.06)	27,580 (22.45)	

BMI, body mass index; NSAIDs, non-steroidal anti-inflammatory drugs; UV, ultraviolet

Values are means ± SD or number (%) unless stated otherwise

### Fish oil supplementation and SLE risk

During a median follow-up duration of 11.57 years (4,516,716.8 person-years), 141 participants developed SLE among fish oil non-users (4.56 per 100,000 person-years), and 68 participants did among fish oil users (4.78 per 100,000 person-years). The overall incidence of SLE was 4.63 per 100,000 person-years, 7.08 per 100,000 person-years in females, and 1.81 per 100,000 person-years in males, respectively, with a female-to-male ratio of 4:1. We used different models to evaluate the association of fish oil supplementation with the risk of incident SLE. In the age- and sex-adjusted model (model 1), no significant association was found between fish oil use and incident SLE (HR: 0.94, 95% CI: 0.70–1.27). This insignificant association remained (HR: 0.84, 95% CI: 0.61–1.15) after adjustment for other general demographic factors, socioeconomic factors, medical history, UV radiation and the use of drugs and other supplements (model 2). The results remained insignificant even after further adjusting for ten types of diet components, or the healthy diet score (model 3 and model 4, Table 2). The detailed Cox regression results of model 4 are presented in Supplementary Table S5.

**Table 2:** Association between fish oil use and risk of SLE among the 390,277 participants

	Fish oil non-users (*n* = 267,448)	Fish oil users (*n* = 122,829)	*p*-value
Number of cases	141	68	
Person-years	3,092,936	1,423,781	
HR (95% CI)			
Model 1	1 (ref)	0.94 (0.70, 1.27)	0.70
Model 2	1 (ref)	0.84 (0.61, 1.15)	0.28
Model 3	1 (ref)	0.86 (0.63, 1.18)	0.36
Model 4	1 (ref)	0.85 (0.62, 1.16)	0.30

CI, confidence interval; HR, hazard ratio

Model 1 adjusted for age and sex; Model 2 adjusted for age, sex, race, location of assessment centers, BMI, education, Townsend deprivation index, smoking status, alcohol drinking status, physical activity, vitamin supplementation use, mineral supplementation use, nonsteroidal anti-inflammatory drugs use, history of hypertension, history of diabetes, history of hyperlipemia and ultraviolet radiation; Model 3 further adjusted for fruits, vegetables, whole grains, refined grains, fish, dairy products, vegetable oil, processed meat, unprocessed meat, and sugar-sweetened beverages based on model 2; Model 4 further adjusted for healthy diet score based on model 2

### Subgroup and sensitivity analyses

**Fig. 2:**
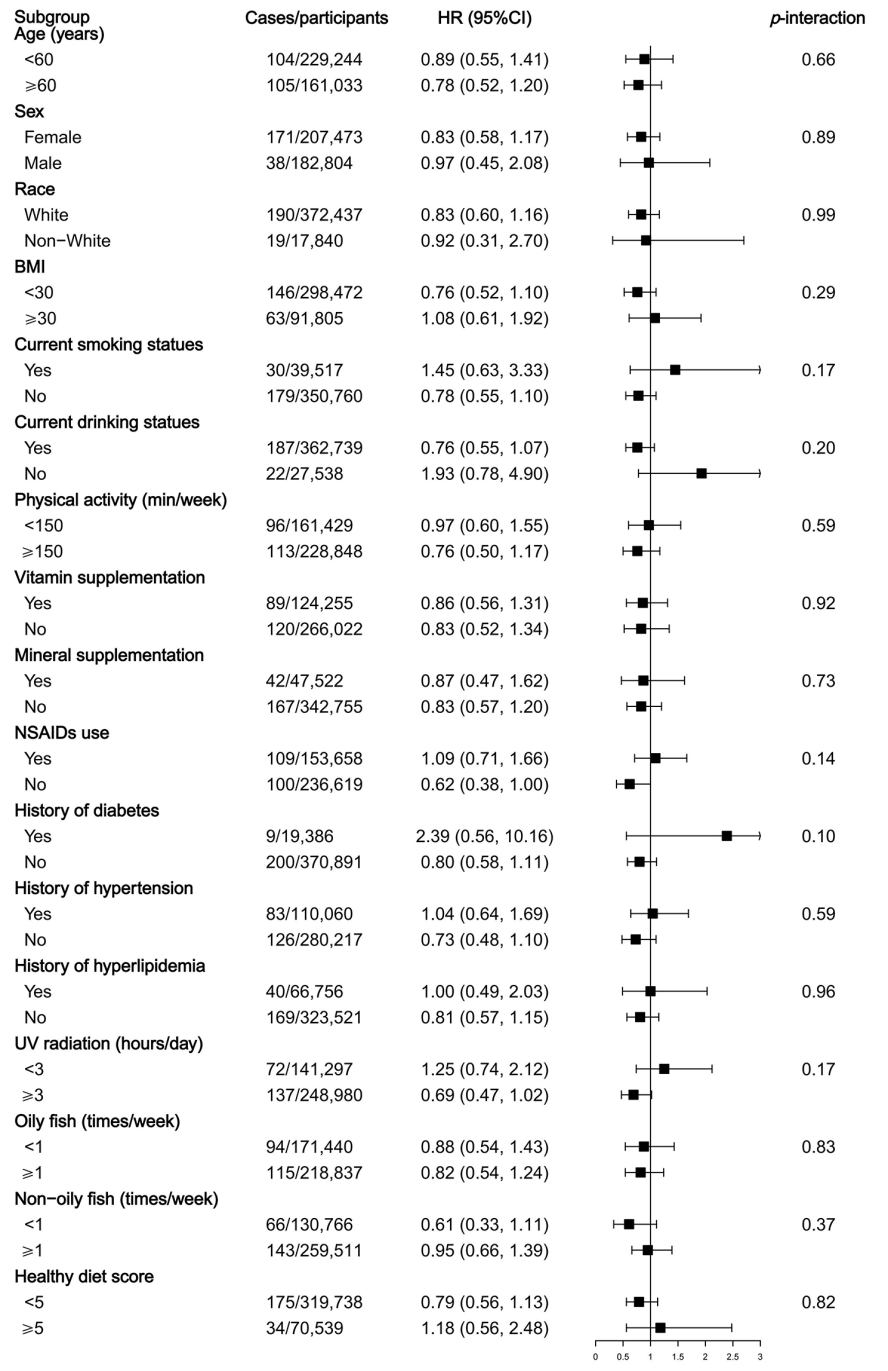
Subgroup analyses for the association between fish oil use and the risk of SLE. Note: BMI, body mass index; CI, confidence interval; HR, hazard ratio; NSAIDs, non-steroidal anti-inflammatory drugs. Forest plots show the multivariable HRs of SLE associated with fish oil use in subgroups. HRs were adjusted for age, sex, race, location of assessment centers, BMI, education, Townsend deprivation index, smoking status, alcohol drinking status, physical activity, vitamin supplementation use, mineral supplementation use, nonsteroidal anti-inflammatory drugs use, history of hypertension, history of diabetes, history of hyperlipemia, ultraviolet radiation and healthy diet score

To examine the potential modifications between fish oil use and the risk of SLE, we further conducted subgroup analyses. The results showed no significant interactions when the analyses were stratified by potential risk factors, including age, sex, race, BMI, smoking status, alcohol drinking status, physical activity, UV radiation, vitamin supplementation, mineral supplementation, the use of NSAIDs, history of diabetes, history of hypertension, history of hyperlipemia, oily fish intake, non-oily fish intake, and healthy diet score (Fig. 2). Likewise, no significant association between fish oil use and SLE risk was observed in any subgroup.

**Fig. 3:**
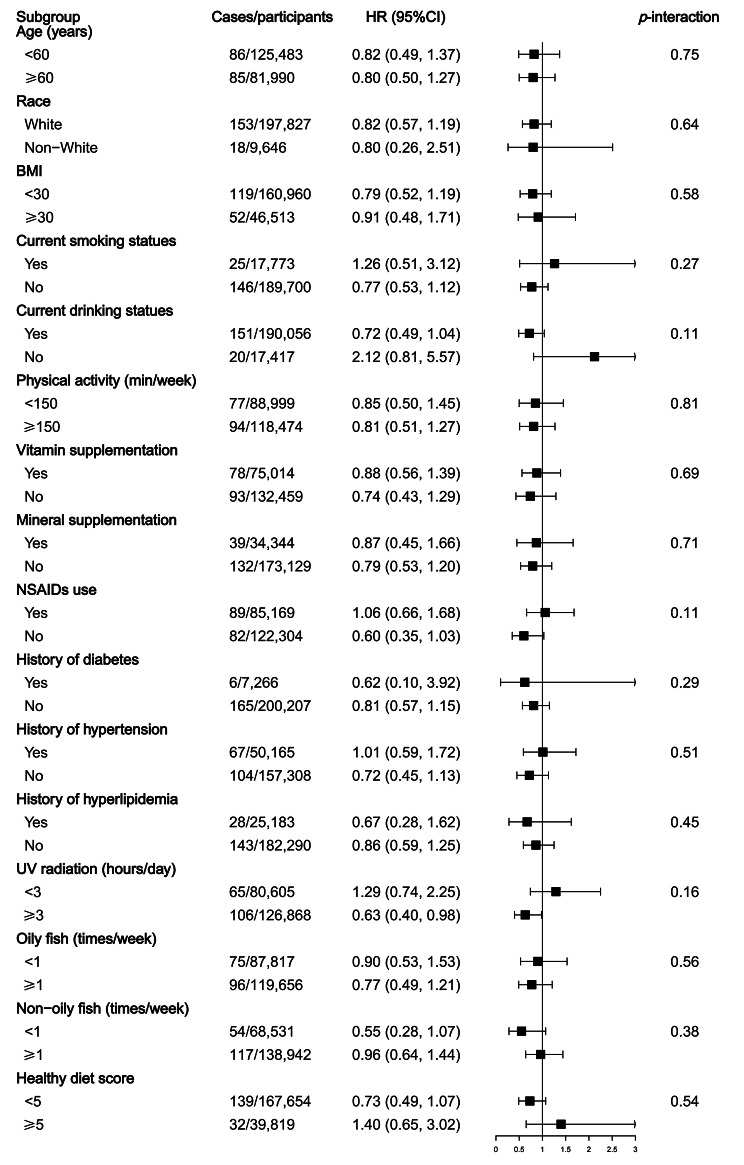
Subgroup analyses for the association between fish oil use and the risk of SLE in female group. Note: BMI, body mass index; CI, confidence interval; HR, hazard ratio; NSAIDs, non-steroidal anti-inflammatory drugs. Forest plots show the multivariable HRs of SLE associated with fish oil use in subgroups. HRs were adjusted for age, sex, race, location of assessment centers, BMI, education, Townsend deprivation index, smoking status, alcohol drinking status, physical activity, vitamin supplementation use, mineral supplementation use, nonsteroidal anti-inflammatory drugs use, history of hypertension, history of diabetes, history of hyperlipemia, ultraviolet radiation and healthy diet score

Furthermore, in view of the female predominance of SLE incidence [31], we conducted subgroup analysis in the female group. The results of the female group were basically consistent with those of all participants, suggesting no significant interactions between fish oil use and potential risk factors in the female group (Fig. 3). Interestingly, an inverse association of fish oil use with SLE risk was observed among 126,868 female participants with UV radiation ≥ 3 h/day (HR: 0.63, 95% CI: 0.40–0.98). However, no significant association was observed after further adjustment for age at menarche, oral contraceptive use, menopause status, hormone-replacement therapy use (and further adjustment for sun protection measures) among females in any subgroup, including participants with UV radiation ≥ 3 h/day (Supplementary Figure S1-2).

In the sensitivity analyses, we found that the relation between fish oil use and SLE risk remained insignificant after excluding 17 participants who developed SLE within the first two years of follow-up or further adjustment for sun protection measures (Supplementary Table S6). Results from sensitivity analysis by adjusting for age at menarche, oral contraceptive use, menopause status, hormone-replacement therapy use (and further adjustment for sun protection measures) in the female group also showed no material change (Supplementary Table S6).

## Discussion

In this prospective cohort study of 390,277 participants, on the whole, we did not observe an association between habitual fish oil supplementation and the risk of incident SLE except in females exposed to prolonged UV radiation, even after adjustment for most of the potential confounders. The results do not support a beneficial effect of fish oil use on SLE risk.

It has been reported that the overall incidence of SLE in the UK was 4.91 per 100,000 person-years and the incidence in females was 5.8 times to that of males [33]. In this study, we reported an overall incidence of 4.63 per 100,000 person-years among 390,277 participants from the UKB. Even though the UKB study included participants aged 40–69 years, which is beyond the peak period of SLE occurrence, the incidence we observed is very close to that reported previously in the UK. In fact, other UKB studies showed that the incidences of inflammatory bowel diseases and ankylosing spondylitis, another two autoimmune diseases susceptible to young and middle-aged populations, were higher than those previously reported [20, 34]. It seems that the incidences observed in the UKB participants are generally higher, possibly attributed to better diagnosis from researchers and more detailed self-reported information from participants during follow-up. The not-low incidence in our study strengthened its statistical power to conclude significant findings, which further supported the insignificant relationship between fish oil supplementation and SLE risk. In addition, the female-to-male ratio of SLE incidence varies with age, peaking with a value of almost 10 during the fourth decade and declining in the next three or four decades to almost 3 for elderly adults [35]. The female-to-male ratio of 4:1 observed in our data can be explained by the fact that the individuals included were between the ages of 40 and 69 at baseline with an average age of 56, when female predominance is less pronounced.

According to our knowledge, our study is the first population-based study to evaluate the association of fish oil supplementation with SLE incidence. A previous animal study demonstrated that dietary fish oil supplementation delayed the onset of SLE in NZB/W F1 mice [21], a commonly used mouse model of SLE that spontaneously develops SLE characteristics as they age [36]. Another animal study showed that dietary ω-3 PUFA (DHA) supplementation prevented the development and progression of SLE in NZB/W F1 mice treated with crystalline silica (cSiO2), a unique model for environment-triggered SLE [37]. Nevertheless, the effect of fish oil or ω-3 PUFAs supplementation on incident SLE in human level has not been reported. Evidence from cross-sectional studies showed that patients with SLE had lower, higher, or similar serum levels of ω-3 PUFAs compared to healthy controls [38–40], and another recent study reported that SLE patients had higher intakes of ω-3 PUFAs than non-SLE individuals [41]. These inconsistent outcomes have made it more challenging to estimate the role of fish oil or ω-3 PUFAs supplementation in SLE incidence. In this study, we found no evidence of an association between fish oil use and SLE risk. What’s more, the sensitivity analyses yielded consistent outcomes, indicating the robustness of our results. A possible explanation for the result is that the etiology and pathogenesis of SLE are complicated, with inflammation being only one part of it. Therefore, fish oil supplementation alone may not be sufficient to lower the risk of SLE in the general population. It filled the blank in direct evidence on whether fish oil use has a protective role in SLE risk and clarified that fish oil supplementation may have no health benefit in terms of lowering the risk of SLE.

It is known that SLE incidence is much higher in females than in males, especially in females of reproductive age (20–40 years), which are likely related to a complex interaction between sex hormones, genetics, epigenetics, and gut microbiota [31, 35]. Evidence from several case-control studies indicated that exposure to UV radiation may be an environmental trigger for SLE incidence [29, 42, 43]. However, a prospective cohort study recently reported insignificantly increased risk of SLE but significantly increased risk of malar rash, a manifestation of acute cutaneous lupus, in females with higher UV exposure [44]. Our subgroup analysis among females stratified by UV radiation showed that fish oil supplementation was associated with a lower risk of incident SLE when female participants were exposed to longer UV radiation (≥ 3 h/day), while there was no significant interactions between fish oil use and UV radiation. UV radiation contributes to increased susceptibility to SLE by inducing inflammation and immune function change via upregulating multiple inflammatory mediators, interacting with the glutathione S-transferase Mu 1 (GSTM1) null genotype that clear reactive oxygen intermediate more slowly and therefore have more oxidative stress, and increasing the expression of nuclear autoantigens on the keratinocyte surface via triggering DNA damage [43, 45]. Plausible mechanisms of ω-3 PUFAs’ anti-inflammatory property include decreasing the generation of inflammatory mediators like prostaglandins (PGs) by competing with ω-6 PUFAs, suppressing pro-inflammatory cytokine production, and inhibiting the proliferation and function of multiple immune cells [16, 46]. Therefore, a possible explanation for our interesting finding is that fish oil supplementation partly blocks the harmful effects of UV radiation in females on SLE incidence. The findings might have public health implications for the prevention of SLE among specific high-risk individuals, females exposed to high UV radiation. Of note, the significant finding should be applied with caution and needs to be confirmed in further studies, as it is based on a subgroup analysis, which can be more prone to chance findings due to multiple testing, and the association turned insignificant after further adjustment for female-related factors and sun protection measures, indicating the protective role is unstable.

The current study has several advantages. Above all, it is a prospective cohort study in a real-world setting with large numbers of participants (up to 390,277) and a long follow-up period (a median of 11.57 years), which provided sufficient statistical power. Besides, we employed almost all sources to identify the incidence of SLE, including primary care, hospital admission, death register, and self-report. In addition, multi-dimensional covariates were fully considered and carefully adjusted to minimize interference from confounding factors. These measures enhanced the validity of our results. Moreover, sensitivity analyses also demonstrated the robustness of our findings.

However, there are also several main limitations in this study. Firstly, we roughly classified participants as fish oil users or fish oil non-users according to their conditions at baseline, regardless of the variability of individuals’ behavior during the long-term study, which might cause misclassification of exposure. Also, the lack of other information on fish oil use, such as the dose, components, duration, and frequency, eliminated the possibility of discovering that some specific pattern of fish oil supplementation might be associated with SLE risk. Secondly, SLE cases were defined through the ICD-10 code in medical records or self-reports, which could introduce misclassification of the outcome if the diagnostic criteria were not uniformly applied or if self-reported diagnoses were inaccurate. Thirdly, we could not further evaluate the relation between circulating ω-3 PUFAs levels and SLE risk due to our limited access to the UKB data, which restricted the implications of our findings on fish oil supplementation and SLE risk. Likewise, we did not include the polygenic risk score of SLE in our analysis because we did not have access to the genetic data of UKB, although it’s known that genetic factors play a certain role in the risk of SLE [5, 47]. In this case, our results may be affected by residual confounding. Also, we were unable to rule out the possibility of other confounders in this observational study, even though a wide range of covariates were carefully adjusted for in our analyses. Fourthly, we used time spent outdoors on summer days to assess UV radiation exposure, which may not be very accurate; however, it was used as a measure of sunlight exposure in a prior study based on UKB [48]. Finally, it is unclear whether our findings can be generalized to a younger or other racial population, as the study participants are 40–69 years at baseline and 95.43% of them are White. Although the populations of reproductive age were absent, we provided information on the older populations, who develop late-onset lupus with a poorer overall outcome and have been less studied before [31]. Future studies should evaluate the correlation between the intake of fish oil and SLE risk among younger and other racial populations to yield further conclusions.

## Conclusions

In conclusion, our results do not support a beneficial role of fish oil supplementation in SLE risk, though fish oil supplementation was inversely associated with the risk of SLE among females with prolonged exposure to UV radiation. The findings have significant implications for formulating recommendations on effective dietary prevention of SLE, but it should be applied with caution and needs to be confirmed in further studies.  

### Electronic supplementary material

Below is the link to the electronic supplementary material.

Supplementary Material 1

## Data Availability

The data are available on request from the UK Biobank website (www. ukbiobank.ac.uk/).

## Abbreviations

BMI: Body mass index
CI: Confidence interval
DHA: Docosahexaenoic acid
EPA: Eicosapentaenoic acid
HR: Hazard ratio
ICD-10: The International Classification of Diseases version 10
NSAIDs: Nonsteroidal anti-inflammatory drugs
PUFAs: Polyunsaturated fatty acids
SD: Standard deviation
SLE: Systemic lupus erythematosus
TDI: Townsend deprivation index
UKB: United Kingdom Biobank
UV: Ultraviolet
