# Supplementary material for: Fish oil supplementation and risk of incident systemic lupus erythematosus: a large population-based prospective study
Source: Nutr J. 2024 Jun 12;23:63. doi: 10.1186/s12937-024-00965-x (PMC11167923; doi:10.1186/s12937-024-00965-x)
Supplement: Supplementary file 1 — Supplementary Material 1 [file 12937_2024_965_MOESM1_ESM.docx]

**Supplementary Material 1**

**Table S1 Summary of missing data among the 461,646 participants**

| **Characteristics** | **No. of missing (%)** |
| --- | --- |
| Age | 0 |
| Sex | 0 |
| Location of assessment centers | 0 |
| Race | 1597 (0.35) |
| Education | 9130 (1.98) |
| BMI | 2800 (0.61) |
| Smoking status | 2575 (0.56) |
| Alcohol drinking status | 1412 (0.31) |
| Physical activity | 30,376 (6.58) |
| Vitamin supplementation | 6013 (1.30) |
| Mineral supplementation | 4411 (0.96) |
| NSAIDs use | 9451 (2.05) |
| History of diabetes | 545 (0.12) |
| History of hypertension | 927 (0.20) |
| History of hyperlipidemia | 7367 (1.60) |
| Ultraviolet radiation | 30,529 (6.61) |
| Fruit | 1843 (0.40) |
| Vegetable | 3987 (0.86) |
| Oily fish | 3652 (0.79) |
| Non-oily fish | 3284 (0.71) |
| Dairy | 956 (0.21) |
| Whole grain | 1115 (0.24) |
| Refined grain | 1321 (0.29) |
| Vegetable oils | 2815 (0.61) |
| Processed meat | 1942 (0.42) |
| Unprocessed red meat | 6035 (1.31) |
| Sugar-sweetened beverages consumer | 5772 (1.25) |
| Healthy diet score | 18,222 (3.95) |
| BMI, body mass index; NSAIDs, non-steroidal anti-inflammatory drugs. | |

**Table S2 Disease definitions used in the UK Biobank study**

| **Disease** | **ICD-9** | **ICD-10** | **Self-reported fields** |
| --- | --- | --- | --- |
| Diabetes | 250 | E10-E14 | 2443(1), 6153(3), 6177(3), 20002(1220, 1222, 1223) |
| Hypertension | 401-405 | I10-I13, I15, O10 | 6150(4), 6153(2), 6177(2), 20002(1065, 1072) |
| Hyperlipidemia | 2720-2724 | E780-E785 | 6153(1), 6177(1), |

Variable definitions constructed using ICD-9, ICD-10 and self-reported data fields with choice-, disease-specific codes between brackets are

shown. Abbreviations: ICD, International Classification of Diseases.

| **Table S3 Diet component definitions used in the UK Biobank study** | | | |
| --- | --- | --- | --- |
| **Components** | **Intake goal** | **Field IDs** | **Amount per serving** |
| Fruit | 3 servings/day | 1309 (pieces fresh fruit/day)  1319 (pieces dried fruit/day) | 1309 – 1 piece 1319 – 5 pieces |
| Vegetable | 3 servings/day | 1289 (tablespoons cooked vegetables/day)  1299 (salad/raw vegetables/day) | 3 heaped tablespoons |
| Whole grains | 3 servings/day | 1438, 1448 (wholemeal/wholegrain bread slices/week) 1458, 1468 (bran/oat/muesli cereal bowls/week) | 1438/1448 – 1 slice/day  1458/1468 – 1 bowl/day |
| Fish | ≥2 servings/week | 1329 (oily fish/week) 1339 (non-oily fish/week) | Once/week |
| Dairy | 2 servings/day | 1408 (cheese/week) 1418 (milk type) | 1408 – 1 piece/day 1418 – 1 glass/day if consumption of any type of milk |
| Vegetable oils | 2 servings/day | 1428 (Flora Pro-Active/Benecol spread) 2654 (Flora Pro-Active/Benecol, soft margarine -, olive oil based -, polyunsaturated/sunflower oil based -, other low/reduced fat spread) 1438 (bread slices/week) | 1 serving/day if in combination with eating at least 2 slices of bread (ID 1438) |
| Refined grains | ≤2 servings/day | 1438, 1448 (white, brown, other bread slices/week)  1458, 1468 (biscuit, other cereals/week) | 1438/1448 – 1 slice/day  1458/1468 – 1 bowl/day |
| Processed meats | ≤1 serving/week | 1349 (processed meat/week or daily)  3680 (age when last ate meat) | 1349 – 1 piece/day 3680 – 0 pieces/day if indicated having never eaten meat |
| Unprocessed meats | ≤2 serving/week | 1359 (poultry/week or day)  1369 (beef/week or day) 1379 (lamb or mutton/week or day)  1389 (pork/week or day) 3680 (age when last ate meat) | 1359-1389 – once/week 3680 – 0 pieces/day if indicated having never eaten meat |
| Sugar-sweetened beverages | Do not drink | 6144 (never consumes drinks containing sugar) | 0 servings |
| Field IDs and serving sizes used per diet component in UK Biobank with available data from the general baseline questionnaire. If participants achieved the intake goal they were given 1 point for the diet component. The total healthy diet score was the sum of all the diet component scores and ranged from 0 to 10. | | | |

| **Table S4 Measuring methods of potential covariates** | | |
| --- | --- | --- |
| **Variables** | **Measuring method**  **(UK Biobank Touchscreen question****naire)** | **Filed codes in UK biobank** |
| Age at menarche | “How old were you when your periods started?” | Field 2714 |
| Oral contraceptive use | “Have you ever taken the contraceptive pill? (include the ‘mini-pill’)” | Field 2784 |
| Menopause status | “Have you had your menopause (periods stopped)?” | Field 2724 |
| Hormone-replacement therapy used | “Have you ever used hormone replacement therapy (HRT)?” | Field 2814 |
| Sun protection measures | “Do you wear sun protection (e.g. sunscreen lotion, hat) when you spend time outdoors in the summer?” | Field 2267 |

| **Table S5 Results of Cox regression of model 4^a^** | | | |
| --- | --- | --- | --- |
| **Variables** | **Case/participant** | **HR (95% CI)** | ***p*-value** |
| Age (years) | 209/390,277 | 1.01 (0.99, 1.03) | 0.157 |
| Sex |  |  |  |
| Female | 171/207,473 | 1 (ref) |  |
| Male | 38/182,804 | 0.25 (0.17, 0.35) | <0.001 |
| Race |  |  |  |
| White | 190/372,437 | 1 (ref) |  |
| Non-White | 19/17,840 | 1.91 (1.13, 3.23) | 0.016 |
| Education |  |  |  |
| Higher | 121/244,644 | 1 (ref) |  |
| Upper secondary | 13/22,477 | 1.06 (0.60, 1.87) | 0.853 |
| Lower secondary | 30/65,764 | 0.76 (0.51, 1.14) | 0.184 |
| Others | 45/57,392 | 1.26 (0.87, 1.82) | 0.226 |
| Townsend deprivation index | 209/390,277 | 1.01 (0.96, 1.06) | 0.628 |
| BMI (kg/m^2^) |  |  |  |
| <18.5 | 0/1,936 | - | - |
| 18.5-24.9 | 74/129,605 | 1 (ref) |  |
| 25-29.9 | 72/166,931 | 0.82 (0.59, 1.14) | 0.243 |
| ≥30 | 63/91,805 | 1.08 (0.75, 1.55) | 0.672 |
| Smoking status |  |  |  |
| Never | 96/215,608 | 1 (ref) |  |
| Previous | 83/135,152 | 1.48 (1.09, 1.99) | 0.012 |
| Current | 30/39,517 | 1.93 (1.27, 2.96) | 0.002 |
| Alcohol drinking status |  |  |  |
| Never | 10/14,678 | 1 (ref) |  |
| Previous | 12/12,860 | 1.61 (0.68, 3.81) | 0.274 |
| Current | 187/362,739 | 1.09 (0.56, 2.12) | 0.796 |
| Physical activity (min/week) |  |  |  |
| <150 | 96/161,429 | 1 (ref) |  |
| ≥150 | 113/228,848 | 0.89 (0.67, 1.18) | 0.421 |
| Vitamin supplementation |  |  |  |
| No | 120/266,022 | 1 (ref) |  |
| Yes | 89/124,255 | 1.35 (0.99, 1.86) | 0.059 |
| Mineral supplementation |  |  |  |
| No | 167/342,755 | 1 (ref) |  |
| Yes | 42/47,522 | 1.27 (0.87, 1.84) | 0.214 |
| NSAIDs use |  |  |  |
| No | 100/236,619 | 1 (ref) |  |
| Yes | 109/153,658 | 1.41 (1.07, 1.87) | 0.016 |
| History of diabetes |  |  |  |
| No | 200/370,891 | 1 (ref) |  |
| Yes | 9/19,386 | 0.67 (0.33, 1.36) | 0.270 |
| History of hypertension |  |  |  |
| No | 126/280,217 | 1 (ref) |  |
| Yes | 83/110,060 | 1.71 (1.25, 2.34) | 0.001 |
| History of hyperlipidemia |  |  |  |
| No | 169/323,521 | 1 (ref) |  |
| Yes | 40/66,756 | 0.95 (0.63, 1.41) | 0.787 |
| UV radiation (hours/day) |  |  |  |
| <3 | 72/141,297 | 1 (ref) |  |
| ≥3 | 137/248,980 | 1.10 (0.82, 1.47) | 0.538 |
| Healthy diet score |  |  |  |
| <5 | 175/319,738 | 1 (ref) |  |
| ≥5 | 34/70,539 | 0.81 (0.56, 1.17) | 0.266 |
| Fish oil use |  |  |  |
| No | 141/267,448 | 1 (ref) |  |
| Yes | 68/122,829 | 0.85 (0.62, 1.16) | 0.298 |

BMI, body mass index; CI, confidence interval; HR, hazard ratio; NSAIDs, nonsteroidal anti-inflammatory drugs; UV, ultraviolet.

a: result of “location of centers” variable are not presented, because there are 22 centers, it would be very lengthy to show.

**Table S6 Results of sensitivity analyses**

|  | **Fish oil non-users** | **Fish oil users** | ***p-*value** |
| --- | --- | --- | --- |
| Sensitive analysis 1^a^ | |  |  |
| Number of cases | 129 | 63 |  |
| Person-years | 3,092,922 | 1,423,776 |  |
| HR (95% CI) | 1 (ref) | 0.85 (0.61, 1.18) | 0.34 |
| Sensitive analysis 2^b^ | |  |  |
| Number of cases | 141 | 67 |  |
| Person-years | 3091591 | 1423271 |  |
| HR (95% CI) | 1 (ref) | 0.84 (0.61, 1.15) | 0.27 |
| Sensitive analysis 3^c^ | |  |  |
| Number of cases | 113 | 56 |  |
| Person-years | 1,578,426 | 771,073 |  |
| HR (95% CI) | 1 (ref) | 0.84 (0.60, 1.19) | 0.39 |
| Sensitive analysis 4^d^ | |  |  |
| Number of cases | 113 | 55 |  |
| Person-years | 1578055 | 770867 |  |
| HR (95% CI) | 1 (ref) | 0.83 (0.58, 1.18) | 0.29 |

CI, confidence interval; HR, hazard ratio.

a: excluding participants who developed SLE during the first two years of follow-up;

b: further adjusted sun protection measures based on model 4;

c: further adjusted for age at menarche, oral contraceptive use, menopause status, hormone-replacement therapy used based on model 4 (in female group);

d: further adjusted for age at menarche, oral contraceptive use, menopause status, hormone-replacement therapy used and sun protection measures based on model 4 (in female group).

.
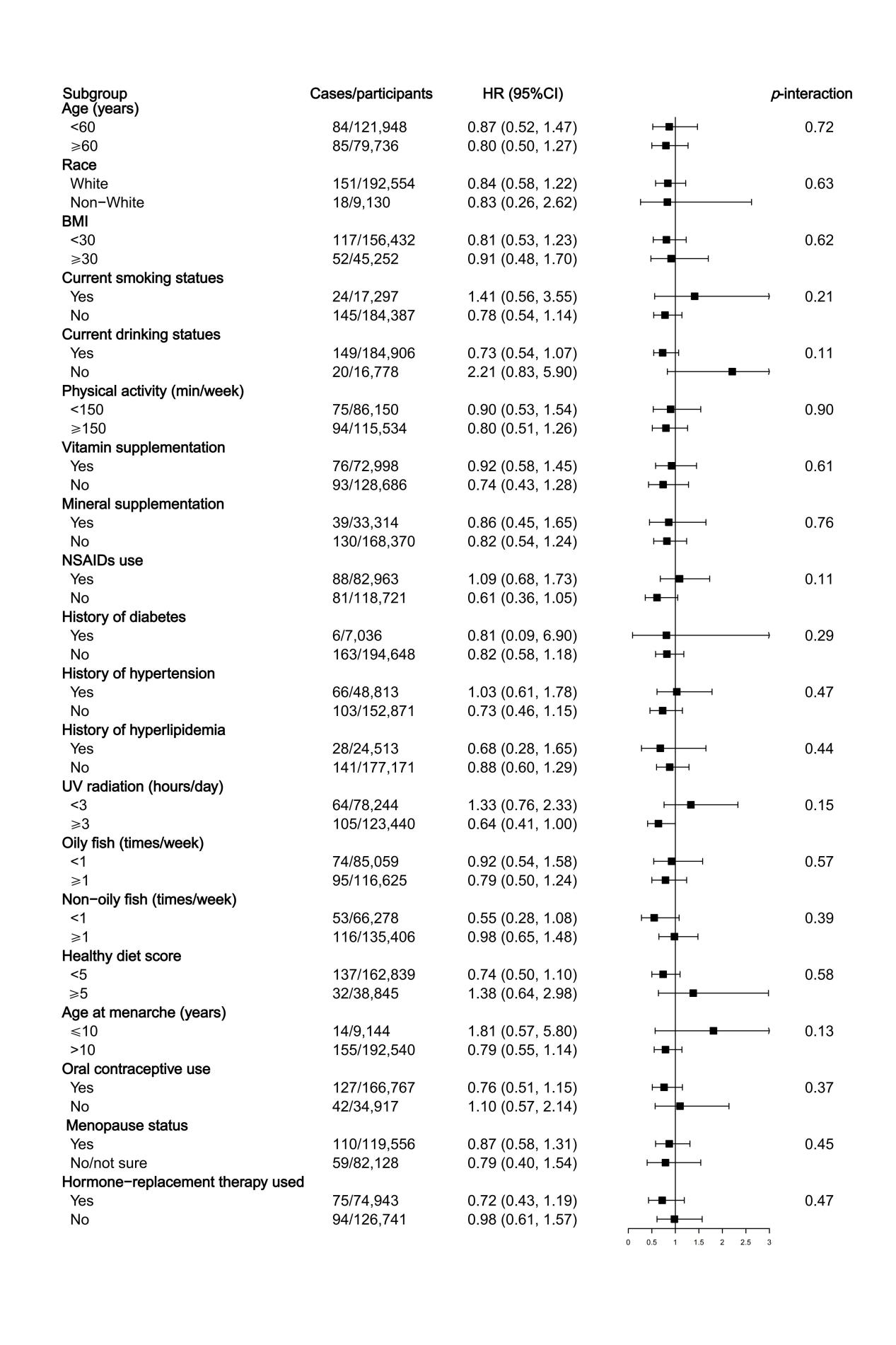


Figure S1 Subgroup analyses for the association between fish oil use and the risk of SLE stratified by potential risk factors in female group, after further adjust age at menarche, oral contraceptive use, menopause status, and hormone-replacement therapy used.


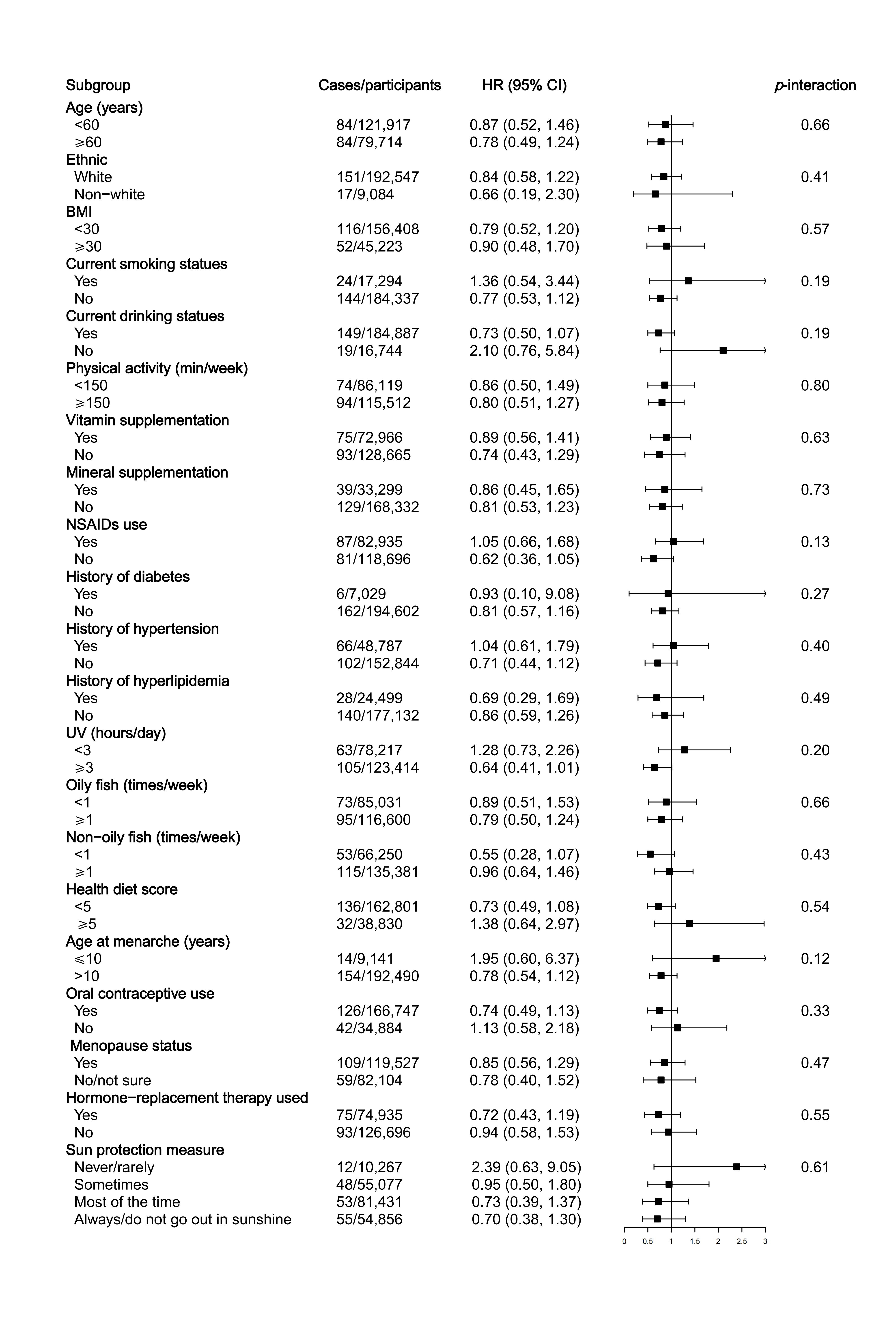


Figure S2 Subgroup analyses for the association between fish oil use and the risk of SLE stratified by potential risk factors in female group, after further adjust age at menarche, oral contraceptive use, menopause status, hormone-replacement therapy used and sun protection measures.
